# Neoadjuvant Chemotherapy for Adults with Osteogenic Sarcoma

**DOI:** 10.1007/s11864-024-01269-2

**Published:** 2024-10-17

**Authors:** Michael J. Robinson, Elizabeth J. Davis

**Affiliations:** Vanderbilt-Ingram Cancer Center, 2220 Pierce Ave, PRB 777, Nashville, TN 37232 USA

**Keywords:** Osteosarcoma, Systemic therapy, Receptor tyrosine kinases, Immunotherapy, Immune Checkpoint Inhibitors, Monoclonal antibodies, Chimeric antigen receptor T cells

## Abstract

Osteosarcoma is the most common primary malignant bone tumor in adolescents and adults. The 5-year survival rate is 65% when localized; however, survival drops dramatically to 10-20% in cases of metastatic disease. Therapy for osteosarcoma saw its first significant advancement in the 1970-80’s, with the introduction of our current standard of care, consisting of the neo/adjuvant treatment regimen methotrexate, doxorubicin (Adriamycin), and cisplatin (collectively referred to as MAP) and surgical resection. Since MAP, development of a better therapeutic approach has stalled, creating a plateau in patient outcomes that has persisted for 40 years. Despite substantial research into a variety of pathways for novel treatment options, clinical trials have not produced sizeable improvements in outcomes. In this article, we discuss our current neoadjuvant standard of care therapy, followed by a review of contemporary therapeutic options, including tyrosine kinase inhibitors (TKIs), immune checkpoint inhibitors (ICIs), monoclonal antibodies (mAbs), and chimeric antigen receptor (CAR) T cells. Lastly, we consider the challenges hindering the success of novel treatment options and future research directions.

## Introduction

Osteosarcoma is the most common malignant bone tumor, with approximately 800-900 new diagnoses in the United States annually [1, 2]. Primary sites of disease occur most commonly at the metaphysis of long bones, with a tendency towards the proximal tibia/fibula, distal femur, and proximal humerus. Adolescents and young adults (AYA) experience the highest incidence of disease, which is thought to coincide with the pubertal growth spurt [3]. A second, smaller, peak occurs among patients around 60 years of age, accounting for approximately 10% of cases and often occurs secondary to Paget’s disease [2]. Overall, a slight male predominance exists, with men affected 1.5 times as often as women [4].

While osteosarcoma demonstrates a heterogenous histology, it is universally derived from mesenchymal stem cells (MSCs) that are directed towards osteoblastic differentiation, with malignant cells producing a mineralized osteoid extracellular matrix [3, 5, 6]. There are several syndromes that confer a predisposition to osteosarcoma, most notably hereditary retinoblastoma and Li-Fraumeni Syndrome [6].

With osteosarcoma, there are both patient and tumor factors that confer an increased risk of mortality, including advanced age, pathologic fractures, appendicular skeleton site of disease (including the pelvis), and tumor size. The most important negative prognostic factor is presence of metastases, which significantly increases mortality [7]. While the 5-year survival rate among patients with localized disease is approximately 65%, it is markedly reduced to 10-20% among patients with metastatic disease [2, 5, 6].

### Evolution of Systemic Therapy

Prior to the introduction of systemic therapy, treatment for osteosarcoma consisted mostly of radical resection (amputation) of the primary tumor site. Unfortunately, surgery alone resulted in more than 80% of patients developing metastatic disease within 6 months after presentation and death often occurred within 2 years of diagnosis [2, 8, 9].

Surgery alone fails to eliminate microscopic disease which results in metastases [1, 9]. Radiotherapy is not typically considered to be a standard for local control, as osteosarcoma is generally regarded to be radioresistant; however, it is considered when tumors are unresectable or when surgical resection confers a high risk of morbidity [6].

Multiple studies have demonstrated the prognostic improvement in patients who received systemic therapy over surgery alone [4, 10]. In a landmark pilot study published in 1979, Rosen et al. determined that the addition of neoadjuvant chemotherapy demonstrated improved survival compared to adjuvant therapy alone. In their study, 31 patients received preoperative systemic therapy with high dose methotrexate (HDMTX), vincristine (VCR), and Adriamycin for three months prior to local control with surgery. This regimen was continued following *en bloc* resection for an additional five months, with the inclusion of cyclophosphamide. After a 4-year follow-up period, patients receiving neoadjuvant therapy demonstrated an overall survival (OS) of 77%, compared to 52% among patients receiving adjuvant therapy alone. The authors theorized that neoadjuvant chemotherapy provided several advantages, most notably early systemic treatment of metastatic microfoci [10]. After this trial, sarcoma centers began incorporating neoadjuvant therapy into practice with similar results. Multi-agent neo/adjuvant chemotherapy improved relapse-free survival to 61%, compared to 11% among controls. During the 1980’s, Link et al. published results from their studies demonstrating that the addition of systemic chemotherapy following surgical resection could generate markedly increased relapse free survival rates among patients with disease localized to an extremity [11, 12].

This practice evolved into what has become the backbone of traditional cytotoxic treatment, which remains largely unchanged to this day [3]. Collectively referred to as MAP, this regimen includes neoadjuvant methotrexate, doxorubicin (Adriamycin), and cisplatin as a standard across America and Europe. [6, 13, 14].

To summarize, the use of neoadjuvant regimens has significantly improved survival and limb salvage rates, as well as reducing disease recurrence and metastasis, resulting in a 5-year OS rate of 65-70% in patients with localized disease. Despite these improvements, patients with metastatic disease continue to demonstrate a significantly inferior survival [13, 15].

## A Contemporary Approach

In the decades since MAP was identified as standard therapy, patient survival has remained relatively stagnant. This is likely due to the challenges associated with osteosarcoma’s significant tumor heterogeneity, variations among tumor microenvironments (TMEs), and low incidence of disease. Given a lack of any significant increase in OS over the last 4 decades, modern therapeutic approaches are needed [16].

The challenges in developing novel neoadjuvant therapy regimens for osteosarcoma involve a lack of distinct targetable antigens [17]. However, recent advancements in the genetic characterization of osteosarcoma has created the potential to explore new treatment opportunities [2].

Prospective contemporary approaches to neoadjuvant therapy include combining cytotoxic therapy with tyrosine kinase inhibitors (TKIs), immunotherapies, and agents targeting cell surface antigens. Experts agree that significant advancements in treating osteosarcoma will not result from intensification of cytotoxic chemotherapy but from alternative and innovative approaches [18].

Another significant challenge in the development of new therapeutic strategies is a lack of clinical trials available to patients at diagnosis. Most trials are designed for patients with recurrent, relapsed, or progressive disease and therefore limit opportunities to explore their efficacy in the neoadjuvant setting.

### Systemic Chemotherapy

As previously mentioned, few significant alterations to the traditional approach of neoadjuvant systemic therapy have been made in the last several decades. However, there is currently one actively enrolling interventional clinical trial evaluating changes to neoadjuvant cytotoxic treatment. This phase II/III trial (NCT05057130) compares doxorubicin/cisplatin to MAP, rationalizing that HDMTX can cause increased adverse events in adults >24 years old. The study is designed as a non-randomized, open-label, parallel comparative study with the primary outcome of overall response rate and secondary outcomes of tumor necrosis and safety. A prior study also evaluated a similar question. In their 2018 study, Wippel et al. compared outcomes between children and young adults who received HDMTX. There was no difference in histologic response rate or metastasis-free survival between the two arms. The median MTX clearance time among patients ≤ 18 years was 79 hours and significantly improved compared to the median clearance time of 120 hours among patients >18 years (*P* < 0.001). Results demonstrated 6 of 8 patients who developed ≤ grade 2 renal insufficiency were greater than 18 years of age, while no grade 3 or greater toxicities were reported among the entire cohort. The authors suggest that proper supportive care practices can mitigate potential concerns of administering HDMTX in patients greater than 18 years of age [19]. Despite this data, prolonged clearance times among older patients may preclude administration of HDMTX.

### Receptor Tyrosine Kinases

Receptor tyrosine kinases (RTKs) are a family of transmembrane glycoproteins involved in extracellular signaling into cells, which regulate cellular differentiation and proliferation. Alterations in RTKs can lead to derangements in cellular growth patterns and proliferation, ultimately leading towards tumorigenesis [20]. TKIs have been evaluated as a potential therapeutic option for bone malignancies, including osteosarcoma.

Regorafenib, cabozantinib, sorafenib, apatinib, and anlotinib have been evaluated in recurrent, relapsed, or metastatic osteosarcoma and demonstrated degrees of efficacy. Regorafenib is a multikinase inhibitor (MTKI) and its antitumor activity in bone sarcomas was evaluated in the REGOBONE study, a double-blind, placebo-controlled, phase II trial. Results revealed that 65% of patients in the treatment arm demonstrated non-progression at 8 weeks. This contrasts with a non-progression rate of 0% in the placebo arm. The regorafenib group also experienced longer median progression free survival (PFS) of 16.4 weeks (95% CI 8.0-27.3) versus 4.1 weeks (95% CI 3.0-5.7) in the placebo group [21, 22].

Cabozantinib is a TKI targeting MET and VEGFR2. Italiano et al. evaluated the efficacy of cabozantinib in a cohort of patients (*N* = 90) with either osteosarcoma (*N* = 45) or Ewing sarcoma (*N* = 45). Following a median follow up time of 31.1 months among the osteosarcoma arm with a final count of 42 assessable participants, 16.7% (7/42) demonstrated a partial response to treatment while 33.3% (14/42) had stable disease. Therapy was well-tolerated with mostly mild adverse events [23]. At the time of this publication, there are two ongoing studies evaluating the potential combination of cabozantinib along with immunotherapy. One is investigating cabozantinib plus atezolizumab, a programmed death-1 (PD-1) receptor inhibitor, in cases of AYA recurrent/metastatic osteosarcoma (NCT05019703), while the other is evaluating cabozantinib plus pembrolizumab (PD-1 inhibitor) (NCT05182164).

Sorafenib is a TKI that targets Raf, Mek, and Erk. Grignani et al. previously investigated the activity of sorafenib in relapsed and unresectable high-grade osteosarcoma. In their study, which included 35 patients, the PFS rate at four months was 46%. Partial response rate was 8% (3/35), and the stable disease rate was 34% (12/35) [24]. These results were encouraging and provided the rationale for evaluating a combination of sorafenib with everolimus (an mTOR inhibitor), suggesting this combination could overcome potential resistance of the mTOR pathway to sorafenib alone. Among 38 patients who received a combination of sorafenib and everolimus, PFS rate at 6 months was 45% (17/38), which was slightly below the prespecified aim of a PFS rate of 50% [25].

Apatinib is a highly selective inhibitor of the vascular endothelial growth factor receptor 2 (VEGFR-2) tyrosine kinase and has been studied as treatment for unresectable, locally advanced, or metastatic osteosarcoma. In a 2018 study, 37 participants with advanced osteosarcoma received apatinib, and the objective response rate (ORR) was 43.24%, while the PFS rate was 56.76% (95% CI, 39.43%–70.84%). These results are similar to other TKIs, such as regorafenib. However, adverse events were severe and did lead to dose reductions and treatment interruptions in 67.57% of cases. This may have been secondary to the increased dose utilized in this trial (750mg) compared to that used in prior studies (500mg). Despite these adverse events, disease response to the intervention led the authors to conclude that apatinib is an effective TKI for treating osteosarcoma [20, 26].

Anlotinib is a MTKI targeting vascular endothelial growth factor receptor (VEGFR), fibroblast growth factor receptor (FGFR), and platelet-derived growth factor receptor (PDGFR). In their 2021 study, Liu et al. evaluated anlotinib as a treatment for unresectable or metastatic bone sarcoma. Results from their study showed that among patients with osteosarcoma, the (ORR) was 7.4% with a median PFS of 4.7 months. Authors suggest these results are similar to most other TKIs along with a comparable side effect profile [27].

Surufatinib is an MTKI, which targets FGFR, colony-stimulating factor 1 receptor (CSF1R), and VEGFR. An upcoming phase II trial (NCT0592649) will be evaluating the impact of surufatinib combined with a three-drug neoadjuvant regimen, consisting of doxorubicin, cisplatin, and ifosfamide as a first-line treatment for osteosarcoma. The trial is a prospective, multi-center, non-randomized (1:1) study with plans to enroll 160 participants up to age 70 years prior to anticipated study completion in 2026. The intervention of interest, surufatinib, builds upon prior studies that suggest it demonstrates efficacy in suppressing osteosarcoma cell migration and invasion [28].

In conclusion, there have been numerous studies of TKI use in relapsed or refractory osteosarcoma. Comparison between trials is limited; however, response and disease control rates seem relatively comparable. Selection of a specific TKI may be driven by cost, availability, or side effect profile. Future studies are needed to compare TKIs head-to-head prospectively as well as to evaluate their utility in the neo/adjuvant setting.

### Immunotherapy

Recent studies have suggested that the TME has a significant impact on chemoresistance patterns by influencing the local immune milieu, which can reduce antitumor activity. Alterations of the TME have the potential to create an imbalance of pro- and anti-inflammatory cell activity that can support tumor progression and metastasis [29, 30]. This phenomenon presents a challenge, and opportunity, for further advancements in osteosarcoma treatment. Potentially, immunotherapy can reduce the TME’s support of chemoresistance by altering or enhancing the immune system to target tumor cells [30, 31]. In this section, we explore several immunotherapeutic strategies, including immune checkpoint inhibitors (ICIs), monoclonal antibodies (mAbs), and chimeric antigen receptor (CAR) T Cells.

#### Immune Checkpoint Inhibitors

ICIs have previously demonstrated anti-tumor activity and are approved for treatment in several types of malignancies. Exploitation of immune checkpoints has the potential to modify the TME in favor of activating an immune response to osteosarcoma. Several recent studies have explored this novel approach to therapy with promising results [32].

The PD-1 receptor is a transmembrane protein and ICI receptor expressed on the surface of a variety of immune cells, including T-cells, macrophages, and monocytes. Its ligands, programmed death-ligand 1 (PD-L1) and programmed death-ligand 2 (PD-L2), are also expressed on macrophages, fibroblasts, T cells, in addition to a variety of tumor cells. Its overexpression on osteosarcoma cells allows for malignant cells to go undetected by T cells, ultimately leading to immune escape by inhibiting T cell mediated cytotoxicity [32, 33]. Prior studies have demonstrated that expression of PD-L1 on osteosarcoma cells is associated with a worse prognosis. Currently, several PD-1 inhibitors, including nivolumab, pembrolizumab, and cemiplimab, in addition to PD-L1 inhibitors, such as atezolizumab, avelumab, and durvalumab are available to treat a variety of malignancies [32]. Researchers have studied the potential therapeutic utility of these drugs in osteosarcoma with mixed results [18, 34–36].

SARC028, a single arm phase II trial, evaluated the safety and efficacy of pembrolizumab in the setting of advanced bone and soft tissue sarcomas. Data from this trial, published in 2017, demonstrated that among 22 patients with osteosarcoma, the partial response rate was 5% (1/22) [36]. In 2021, Boye et al. published results from a single arm phase II trial, which also evaluated the use of pembrolizumab among a cohort of 12 patients with advanced osteosarcoma. Results demonstrated no significant anti-tumor activity or clinical benefit [35].

Anopen-label, single center, phase II clinical trial (NCT0429451), was designed to evaluate the efficacy and safety of camrelizumab, a PD-1 inhibitor, in combination with neoadjuvant chemotherapy in the treatment of osteosarcoma. The trial was initiated in 2019 and designed to determine the extent of cell necrosis when used in combination of a four-drug regimen (doxorubicin, cisplatin, methotrexate, and ifosfamide). Results have not yet been published.

CTLA-4 (cytotoxic T-lymphocyte antigen-4) is a glycoprotein co-inhibitory receptor involved in T-cell activation, which is expressed in osteosarcoma cells, among other types of malignancies [37, 38]. Ipilimumab, a CTLA-4 inhibitor, is currently FDA approved for use in advanced melanoma, and its application in the treatment of osteosarcoma is currently under investigation [32].

Combining PD-1 and CTLA-4 inhibitors may provide synergistic capabilities, improving anti-tumor efficacy compared to their use individually [37]. Results from a study conducted by Lussier et al. found success (50% control of tumor spread) when utilizing this combined approach for metastatic osteosarcoma in murine models [38, 39]. The results of a phase II trial evaluating the efficacy and safety of nivolumab, a PD-1 inhibitor, alone or in combination with ipilimumab in patients with advanced sarcomas did not demonstrate a significant response. Objective responses were noted in 5% (2/38) and 16% (6/38) in the nivolumab and ipilimumab arms, respectively. Of note, only one patient with osteosarcoma was randomized to each arm [40].

In their 2022 literature review of prior studies focusing on the utility of ICIs, Wen et al. reported that patients with osteosarcoma demonstrated a low response rate to this treatment modality. In their review, the authors evaluated prior studies that focused on ICIs targeting CTLA-4, PD-1, and PD-L1, either as mono- or dual therapy. While there were limitations, including few clinical trials and small cohorts, they concluded that ICIs were unable to demonstrate significant anti-tumor activity. Proposed explanations as to osteosarcoma’s resistance to ICIs included muted tumor antigen presentation (necessary for tumor infiltrating lymphocytes) and an intra-tumoral immunosuppressive microenvironment [32].

In summary, current literature investigating the utility of ICIs is limited. Given the heterogeneous nature of osteosarcoma, small sample sizes may miss a signal of activity. Further studies are needed better understand the role of ICIs in the treatment of osteosarcoma.

#### Monoclonal Antibodies and Chimeric Antigen Receptor (CAR) T Cells

Targeting cell surface antigens on osteosarcoma cells is another potential therapeutic strategy. Prior iterations have been implemented in the form of mAbs, CAR T cells, and antibody-drug conjugates (ADCs), and have been used with success in hematologic malignancies [41]. A significant hurdle in targeting cell surface antigens on osteosarcoma cells has been identifying distinct targets. Ideally, these would include cell surface antigens that are highly expressed on most osteosarcoma cells yet spare normal and healthy tissue [9, 41–43]. Two surface antigens have been commonly identified for potential targeting, including disialoganglioside (GD2) and human epidermal growth factor receptor-2 (HER2) [44].

There have been a few studies evaluating mAbs in the treatment of osteosarcoma [33]. In 2012, Ebb et al. published a phase II trial assessing the use of Trastuzumab (a mAb directed at HER2), in combination with cytotoxic therapy among a cohort of newly diagnosed patients with metastatic osteosarcoma. Patients with and without HER2 positive tumors received cisplatin, doxorubicin, methotrexate, ifosfamide, and etoposide. Only tumors with HER2 overexpression were treated with Trastuzumab. Results of the study demonstrated no significant difference in either 30-month event free survival (EFS) or OS between both treatment groups (32% and 59% among the HER2-positive group vs. 32% and 50% in the HER2-negative group) [45].

Another study evaluated the role of the immunoglobulin G1 (IgG1) antibody, Cetuximab, which inhibits the extracellular domain of epidermal growth factor receptor (EGFR), preventing downstream events critical for cell proliferation. Results demonstrated 1 of 21 patients with EGFR+ tumors achieved PFS at 4-months (vs 3 of 15 among EGFR- tumors), leaving authors to conclude that there was no significant activity among patients with EGFR+ osteosarcoma [46].

Despite these results, there remains optimism that mAbs can have a positive impact in the treatment of osteosarcoma given their efficacy in other malignancies. Current and future studies are focused on the potential impact and efficacy of mAbs in cases of recurrent or refractory disease [33].

While CAR T cell therapy has been successful in treating advanced hematologic malignancies, its utility is investigational in sarcomas. The mechanism of CAR T therapy offers an opportunity to selectively target tumor-associated antigens, by engineering antigen specific receptors on T cells to bind tumor cells and facilitate cytotoxicity [33, 41]. Just as HER2 and GD2 are appealing mAb targets, they are attractive as CAR T targets as well [33, 44, 47] given their overexpression on osteosarcoma cells [43, 48].

In a 2020 study evaluating the efficacy of CAR T cells targeted towards GD2 on osteosarcoma cell lines, Chulanetra et al. demonstrated effective killing activity when therapy was synergistically combined with doxorubicin, laying the groundwork for future in vivo trials [49]. Currently, there is one phase I study (NCT03721068) utilizing CAR T-cells modified to target the GD2 antigen in cases of relapsed/refractory neuroblastoma and osteosarcoma.

Prior studies have demonstrated the successful application of CAR T cells in pre-clinical and in vitro models [48, 50]. For example, in 2011 Rainusso et al. demonstrated the successful application of anti-HER2-CAR T cells against drug resistant tumor initiating cells and osteosarcoma cell lines [51].

The results of a phase I/II clinical trial, published in 2015, evaluating the use of HER2 specific CAR T cells in recurrent or refractory osteosarcoma, demonstrated no complete responses to therapy. However, 4 of 17 patients did demonstrate stable disease for 12 weeks to 14 months, 3 of which did not require additional therapy and subsequently had their residual tumor removed. The authors suggested the need for additional investigation and potential pairing of CAR T therapy with other treatment modalities, such as immunotherapies [47].

At the time of this publication, a phase I trial (NCT04995003) is recruiting patients to evaluate the safety of HER2 targeted CAR T cell therapy in combination with an ICI (pembrolizumab or nivolumab), in patients with HER2+ sarcoma.

A potential challenge to the successful implementation of CAR T therapy is the TME in supporting growth and proliferation of osteosarcoma cells. The TME is complex with many intricate pathways leading towards both the promotion and elimination of tumor cells [33, 41]. However, early results from studies evaluating the preclinical efficacy of CAR T therapy for osteosarcoma are reassuring. Many previous models evaluating CAR T utilize familiar antigen targets, such as HER2 and GD2. Alternatively, antigens such as B7-H3 and ALPL-1 are now also being explored to broaden the potential for unique therapeutic targets [50, 52, 53]. Continued discovery of novel targets and improved delivery of CAR T (such as CAR design and supportive care strategies) could play a critical role in overcoming the challenges posed by the TME and thus generate improved survival outcomes [50, 54].

## Summary

Over the last several decades, there have been numerous therapeutic advancements in cancer treatment. Despite this, treatment for localized osteosarcoma is unchanged, and survival rates have plateaued. Future studies should focus on novel therapeutic agents and combination approaches. Clues to identifying new drugs may lie within the TME. Given the rare nature of osteosarcoma, clinical trials should focus on multi-institution collaboration and include correlative studies.

## Key References

- Meltzer PS, Helman LJ. New Horizons in the Treatment of Osteosarcoma. N Engl J Med. 2021;385(22):2066-76. 10.1056/NEJMra2103423. PubMed PMID: 34818481.
  - This reference is of outstanding importance because it provides a historical view of treatment modalities and outcomes for osteosarcoma. It also outlines currently goals of treatment. Furthermore, it describes how the use of genomics and immunotherapy are shifting the paradigm of the traditional approach to treatment of osteosarcoma.
- Wood GE, Graves LA, Rubin EM, Reed DR, Riedel RF, Strauss SJ. Bad to the Bone: Emerging Approaches to Aggressive Bone Sarcomas. Am Soc Clin Oncol Educ Book. 2023;43:e390306. 10.1200/EDBK_390306. PubMed PMID: 37220319.
  - This reference is of importance because it explores potential opportunties and challenges for novel therapeutic options for osteosarcoma.
- Wen Y, Tang F, Tu C, Hornicek F, Duan Z, Min L. Immune checkpoints in osteosarcoma: Recent advances and therapeutic potential. Cancer Lett. 2022;547:215887. Epub 20220819. 10.1016/j.canlet.2022.215887. PubMed PMID: 35995141.
  - This reference is of outstanding importance because it provides a thorough description of how immune checkpoint inhibitors can be incoporated into treatment for osteosarcoma. It highlights well studied immune checkpoints and cites studies evaluating their safety and efficacy.
- Park JA, Cheung NV. Promise and Challenges of T Cell Immunotherapy for Osteosarcoma. Int J Mol Sci. 2023;24(15). Epub 20230807. 10.3390/ijms241512520. PubMed PMID: 37569894; PubMed Central PMCID: PMC10419531.
  - This reference is of outstanding importance because it describes areas of prior exploration and opportunities for advancement with regard to T cell immunotherapy. Also, it provides details of how osteosacoma poses specific challenges to the use of this novel therapy.
- Zhu J, Simayi N, Wan R, Huang W. CAR T targets and microenvironmental barriers of osteosarcoma. Cytotherapy. 2022;24(6):567-76. Epub 20220219. 10.1016/j.jcyt.2021.12.010. PubMed PMID: 35193828.
  - This reference is of importance because it describes the potential application of chimeric antigen receptor T cells in the treatment of osteosarcoma and provides updates on research activities utilizing this modality.

## Data Availability

No datasets were generated or analysed during the current study.
